# First evidence of lymphatic filariasis transmission interruption in Cameroon: Progress towards elimination

**DOI:** 10.1371/journal.pntd.0005633

**Published:** 2017-06-29

**Authors:** Hugues C. Nana-Djeunga, Magellan Tchouakui, Guy R. Njitchouang, Jules B. Tchatchueng-Mbougua, Philippe Nwane, André Domche, Jean Bopda, Stève Mbickmen-Tchana, Julie Akame, Ann Tarini, Emilienne Epée, Benjamin D. Biholong, Yaobi Zhang, Jean J. Tougoue, Achille Kabore, Flobert Njiokou, Joseph Kamgno

**Affiliations:** 1 Centre for Research on Filariasis and other Tropical Diseases, Yaoundé, Cameroon; 2 Parasitology and Ecology Laboratory, Department of Animal Biology and Physiology, Faculty of Science, University of Yaoundé 1, Yaoundé, Cameroon; 3 Helen Keller International, Yaoundé, Cameroon; 4 Ministry of Public Health, Yaoundé, Cameroon; 5 Helen Keller International, Regional Office for Africa, Dakar, Senegal; 6 RTI International, Washington, D.C., United States of America; 7 Faculty of Medicine and Biomedical Sciences, University of Yaoundé 1, Yaoundé, Cameroon; Universidade Federal de Minas Gerais, BRAZIL

## Abstract

**Background:**

Lymphatic filariasis (LF) is among the 10 neglected tropical diseases targeted for control or elimination by 2020. For LF elimination, the World Health Organization (WHO) has proposed a comprehensive strategy including (i) interruption of LF transmission through large-scale annual treatment (or mass drug administration (MDA)) of all eligible individuals in endemic areas, and (ii) alleviation of LF-associated suffering through morbidity management and disability prevention. In Cameroon, once-yearly mass administration of ivermectin and albendazole has been implemented since 2008. The aim of this study was to assess progress towards the elimination goal, looking specifically at the impact of six rounds of MDA on LF transmission in northern Cameroon.

**Methodology:**

The study was conducted in the North and Far North Regions of Cameroon. Five health districts that successfully completed six rounds of MDA (defined as achieving a treatment coverage ≥ 65% each year) and reported no positive results for *Wuchereria bancrofti* microfilariaemia during routine surveys following the fifth MDA were grouped into three evaluation units (EU) according to WHO criteria. LF transmission was assessed through a community-based transmission assessment survey (TAS) using an immunochromatographic test (ICT) for the detection of circulating filarial antigen (CFA) in children aged 5–8 years old.

**Principal findings:**

A total of 5292 children (male/female ratio 1.04) aged 5–8 years old were examined in 97 communities. Positive CFA results were observed in 2, 8 and 11 cases, with a CFA prevalence of 0.13% (95% CI: 0.04–0.46) in EU#1, 0.57% (95% CI: 0.32–1.02) in EU#2, and 0.45% (95% CI: 0.23–0.89) in EU#3.

**Conclusion/Significance:**

The positive CFA cases were below WHO defined critical cut-off thresholds for stopping treatment and suggest that transmission can no longer be sustained. Post-MDA surveillance activities should be organized to evaluate whether recrudescence can occur.

## Author summary

Lymphatic filariasis (LF) affects more than 120 million people worldwide, and is considered the second leading cause of permanent and long-term disability. In response to the important burden of this disease, the World Health Organization (WHO) elaborated a strategic plan to eliminate LF as a public health problem through annual preventive chemotherapy (PC), repeated for at least six years, and reaching at least 65% of the population at risk. To date, about 5.63 billion cumulative treatments have been delivered since 2000, and more than 300 million people no longer require PC thanks to successful implementation of the WHO strategy. In Cameroon, PC for LF has been implemented since 2008. The aim of this study was to assess whether the transmission of LF has been interrupted. Cross-sectional surveys were conducted in three evaluation units (EU) in northern Cameroon. The LF prevalence observed in each of these EU was lower than the threshold of infection below which transmission is likely no longer sustainable, suggesting that the transmission of LF has been interrupted in the study area.

## Introduction

Lymphatic filariasis (LF) is among the most widespread neglected tropical diseases. In the mid-1990s, it was reported that about 1.4 billion people were exposed to the disease worldwide, of whom 120 million were infected and more than 40 million disfigured by the disease [1].

One of the core resolutions of the 50^th^ World Health Assembly held in 1997 was to eliminate LF as a public health problem (resolution WHA50.29). To address this global concern, the World Health Organization (WHO) proposed a comprehensive elimination strategy including (i) transmission interruption in endemic communities (so-called mass drug administration or MDA strategy), and (ii) implementation of interventions to prevent and manage LF-associated disabilities (so-called morbidity management and disability prevention or MMDP strategy) [2]. The Global Programme to Eliminate Lymphatic Filariasis (GPELF) was launched in 2000, by the WHO, to elaborate specific plans and coordinate control efforts to reach this ambitious goal. In the MDA strategy, LF must be mapped and preventive chemotherapy (PC) implemented to treat the entire eligible population (areas where prevalence of antigenaemia is ≥ 1%). In areas where onchocerciasis is endemic and where *Wuchereria bancrofti* prevails, the recommended PC is a single dose of a bi-therapy (150 μg/kg of body weight ivermectin in combination with 400 mg albendazole), administered once yearly [3,4]. Since this treatment is not macrofilaricidal, adult worms can remain viable for about six years and the delivery of several rounds of MDA appeared crucial. It is now accepted that annual MDA should be repeated for at least 5 years at adequate levels of coverage, estimated to be at least 65% of the total population in endemic areas (“effective” MDA), to ascertain that the level of infection in the community will be reduced to levels below which transmission cannot be sustained, even after MDA has been stopped [5]. Recent estimates of the impact of MDA during the past 13 years revealed that more than 96 million LF cases were prevented or cured, although as many as 36 million cases of hydrocele and lymphedema remain [1]. However, data reporting interruption of LF transmission are scanty, especially in Sub Saharan Africa where the disease represents one-third of the global burden [6].

Cameroon is known to be endemic to onchocerciasis [7,8] and bancroftian filariasis [9,10], and MDA against LF have been implemented since 2008. Indeed, ivermectin and albendazole have been distributed by community drug distributors (CDDs) following the community directed treatment with ivermectin (CDTI) approach. This strategy has already been implemented 15–20 years earlier to fight against onchocerciasis. As such, the strategy was already well mastered by CDDs and was ongoing smoothly at the time MDAs against LF were implemented, following a door-to-door approach. This study aimed at assessing whether the transmission of LF has been successfully halted in areas where six MDA rounds have already been delivered.

## Methods

### Survey areas and populations

**Fig 1:**
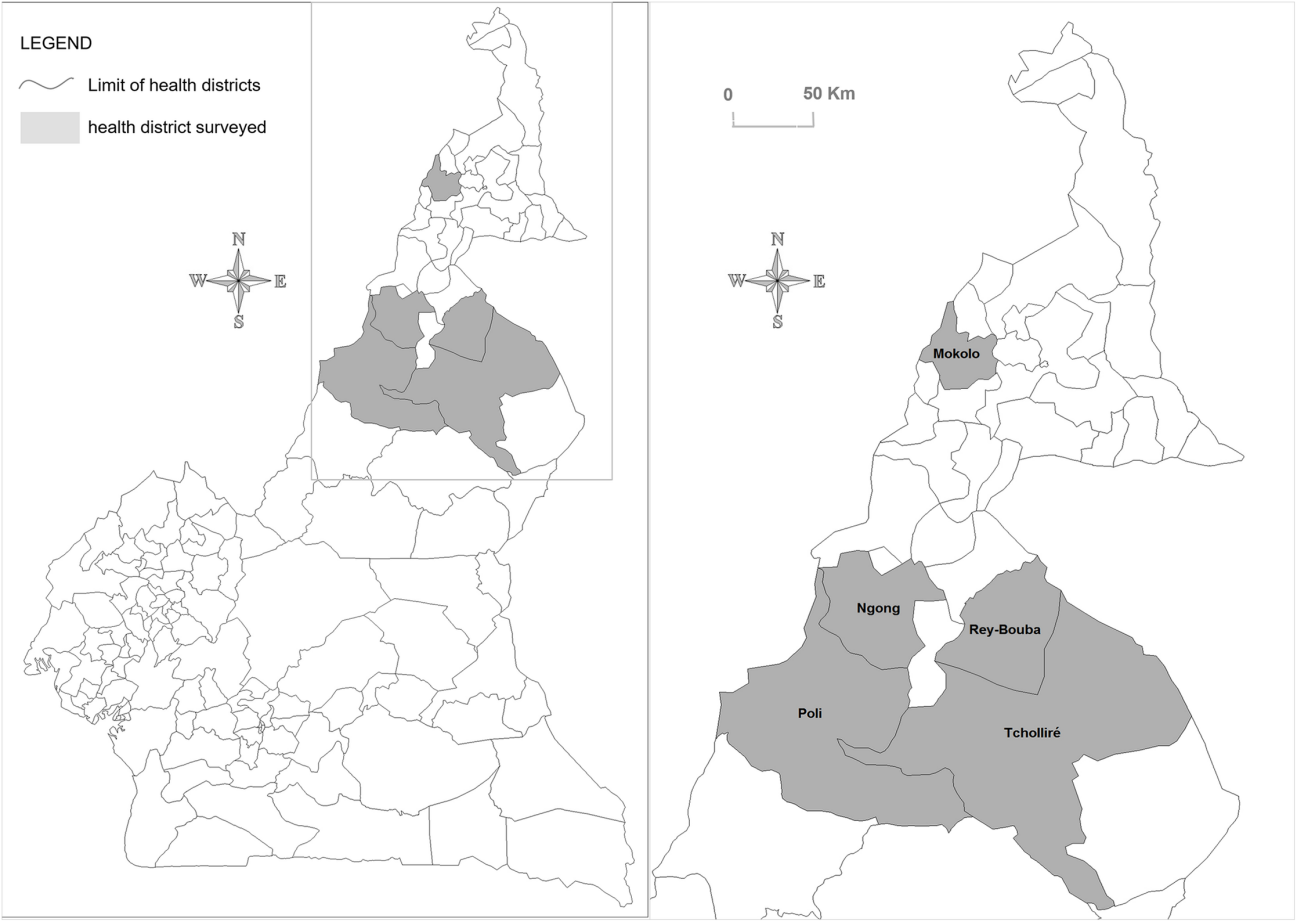
Map showing implementation (IUs) surveyed in the North and Far North Region of Cameroon. The grey zones indicate the IUs surveyed and the blue lines indicate the delineation of evaluation units (EUs).

This study was carried out in 2014 in the North and Far North Regions of Cameroon, situated between latitudes 7° and 12°N, and longitudes 12° and 16°E. Five health districts or implementation units (Ngong, Poli, Tcholliré, Rey-Bouba in the North Region and Mokolo in the Far-North Region), with rural to semi-urban settings, were included in this study. These implementation units (IU) were organized into three evaluation units (EU) (Fig 1) according to the criteria described in the WHO monitoring and evaluation manual [2]. In 2014, the population of each of these two Regions was estimated to two millions, children aged 6–7 years old representing about 10% of the general population [11].

### Study design

A cross sectional study was carried out following the recommendations described in the WHO manual for national elimination programs [2].

### Pre-requisites and eligibility of implementation / evaluation units

**Fig 2:**
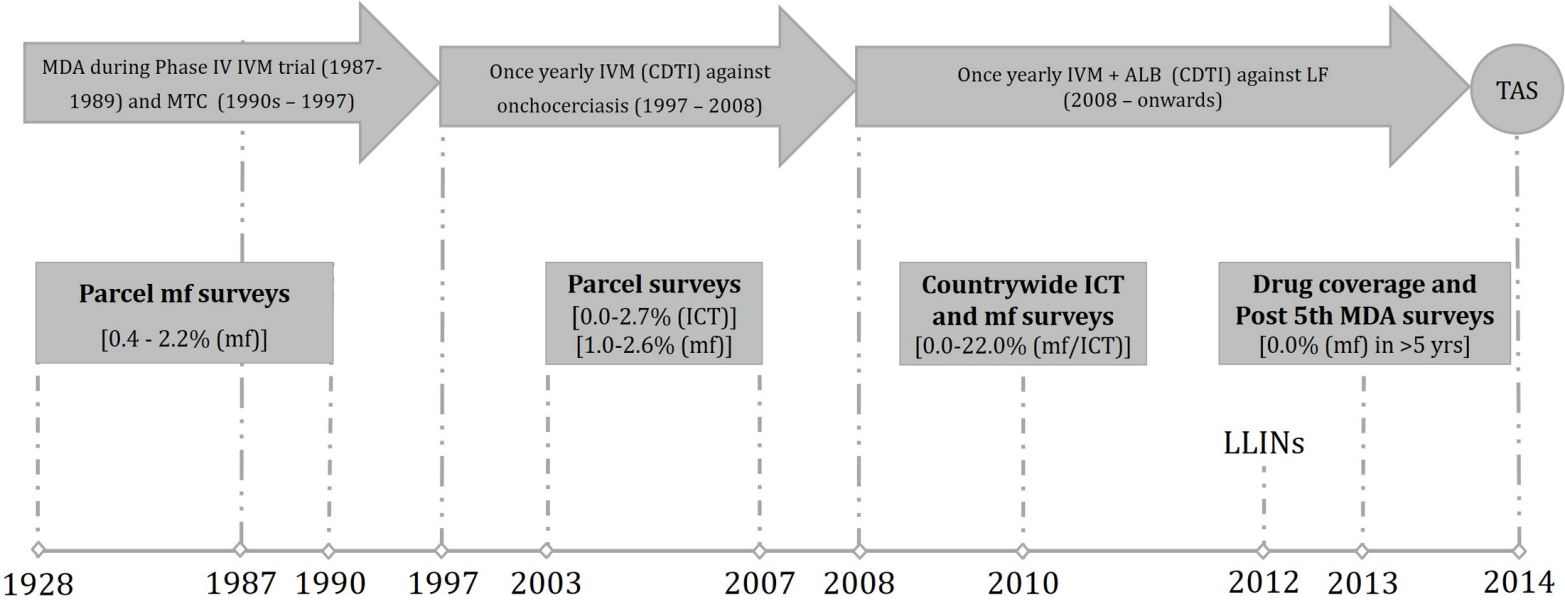
Comprehensive timeline of the programmatic steps taken in the lymphatic filariasis (LF) elimination process in Cameroon. Green boxes indicate surveys (both mapping and impact assessment) and whitish boxes or arrows indicate interventions. ICT: immunochromatographic test; CDTI: community directed treatment with ivermectin; MTC: mobile team campaign; mf: microfilaria; IVM: ivermectin; ALB: albendazole; MDA: mass drug administration; LLINs: long lasting insecticidal nets; TAS: transmission assessment survey.

The flow chart below (Fig 2) describes the different steps taken in the LF elimination process, thus conferring the eligibility of the targeted implementation units to the transmission assessment survey step.

#### Existing data and mapping

LF was known to be endemic in Cameroon since the 1900s thanks to some informative but parcel data from point microfilariaemia surveys (Fig 2). These data had shown that LF was mostly endemic in the northern (North and Far North Regions) Cameroon, with prevalence up to 22% [10]. In 2007, *W*. *bancrofti* circulating filarial antigen (CFA) was detected in 2.7% and 6.9% of individuals tested in four health districts in the Far North using ICT and ELISA, respectively [12], and mf prevalence ranging from 1% to 2% were found during night blood surveys for sentinel site identification in the North and Far North Regions (Ntep, personal communication). In 2009–2010, a nationwide mapping of lymphatic filariasis was organized following WHO recommendations [2], and prevalences ranging from 0 to 20% were found [9].

#### MDA

Since 1987, mass ivermectin distributions, either by mobile teams or through community directed interventions, have been organized over the country in the framework of river blindness control. From 2008 onwards, control efforts are ongoing through once-yearly preventive chemotherapy based on ivermectin and albendazole combination. Due to logistical constraints, health districts (implementation unit, IU) are gradually included in the treatment plan. In 2013, five health districts of the North (Ngong, Poli, Tchollire and Rey-Bouba) and Far North (Mokolo) Regions completed their fifth MDA round, and were therefore eligible to post fifth MDA survey aiming at assessing the impact of MDA on LF infection.

#### Coverage survey

Each of the eligible IU had already completed at least five rounds of annual MDA. After each treatment campaign, drugs’ (ivermectin + albendazole) coverage was reported by the health system at the district level, via the compilation of treatment data from CDDs registers. Indeed, these data showed that reported treatment coverage were in general greater than 65% of the total population (S1 Table), and treatment coverage surveys conducted in 2013 in some of these IU to check whether these reported treatment coverages were accurate confirmed that drug coverages generally exceeded 65% [13].

#### Post 5^th^ MDA survey

A cross sectional survey was conducted in sentinel sites, selected before MDA, where baseline microfilariaemia prevalence was collected for further follow-up of impact of mass treatments on LF endemicity. A comprehensive description of the basic characteristics of sentinel sites are provided in the WHO LF TAS manual [2]. Although no strict rule on the number of sentinel site per IU was edited, it is recommended that a minimum of one sentinel site will be chosen for each 1,000,000 population in the IU. Since the population of the IU considered in this study were relatively small (all between 200,000 and 400,000), it was accepted that one community will be chosen to service as sentinel site in each of the IUs. In these sentinel sites where the likelihood of transmission based on previous surveys were among the highest, a convenient sample of at least 300 individuals, aged 5 years and over, was constituted in each of the five eligible IU, following the WHO recommendations [2]. During this assessment of the impact of five years MDAs in sentinel sites, a total of 1497 individuals (Median age: 16 years old) were examined among which 48% were males. Although 33 hydrocele cases (1.8%; 95% CI: 1.3–2.5) and 2 elephantiasis of lower limb cases (0.1%; 95% CI: 0.0–0.4) were detected during the survey, none of the individuals examined was tested positive for *W*. *bancrofti* microfilariae in night blood smears [14]. Therefore these five IUs were qualified for transmission assessment survey (TAS).

### Transmission assessment survey (TAS)

#### Survey type, target population and sample size

The sampling method used in the evaluation units (EU) was intended to be based on lot quality assurance sampling (LQAS), depending upon factors such as the net primary school enrolment rate in each EU, the target population size, the number of clusters (schools or enumeration areas), and the vector and parasite species. The general design of the TAS was performed using the Survey Sample Builder (SSB), a Microsoft Office Excel based-tool developed by the NTDs Support Centre (http://www.ntdsupport.org/resources/transmission-assessment-survey-sample-builder). Since the net primary-school enrolment ratio was <75% (~60% according to the National Institute of Statistics [15]) in the study area, a community-based cluster survey of children aged 6–7 years old was used. Children from this age group were chosen since they had lived most of their life during the course of MDA, and a positive CFA would likely indicate an ongoing transmission. In addition to the survey design, the SSB was also helpful by calculating automatically the sample size of each EU based on information provided (primary vector species, population of children aged 6–7 years old, total number of EAs, and anticipated non response rate) (S2 Table). The SSB then generated a minimum sample size of 1552 in the EU#1, 1556 in the EU#2 and 1556 in the EU#3, to be collected in 30, 34 and 33 enumeration areas (EAs), respectively. It is worth to mention that, according to WHO guidelines [2], an EA is the smallest area for which census population results are available, and in Cameroon, EAs are equivalent of communities. To randomize household selection in each community or EA, the SSB calculated the sampling interval by dividing the total population of the EU by the number of clusters. Random numbers were then generated by the SSB to be used for the selection of EAs or communities from a list of all communities in each EU beforehand numbered according to geographical proximity. Finally, the sampling fraction (inverse of the sampling interval) was also computed automatically by the SSB to provide the proportion of children to be surveyed per household in each EA to reach the sample size.

#### Diagnostic tools and data collection

*Wuchereria bancrofti* circulating antigen was searched using a rapid-format card test, the immunochromatographic test (ICT) (Alere Inc., Scarborough, USA) [16,17], adhering to the manufacturer’s instructions. The survey was carried out during holidays, thus facilitating the enrollment of children in the communities. Sampling was carried out by skilled laboratory technicians, and training in the use of the ICT cards to detect *W*. *bancrofti* antigen as well as data recording was done for standardization purpose. Besides the antigenaemia data, socio-demographic data (age, sex, community of residence, health area and health district) of each enrollee was also collected.

#### Critical cut-off threshold

This is the threshold of infection prevalence below which transmission is likely no longer sustainable, even in the absence of control interventions. The WHO estimates this threshold by the number of antigen-positive or antibody-positive cases. In the design of the present survey, the critical cut-off value generated by the SSB was 18 for each of the three EUs evaluated.

### Data entry and analysis

All relevant data for this study were recorded into a purpose-built Microsoft Access database and subsequently exported into PASW Statistics version 18 (SPSS Inc., Chicago, IL, USA) for statistical analyses. The prevalences of infection were expressed as the percentage of infected children (harboring CFA) among the total number of children examined; the 95% confidence interval (CI) was calculated using the Wilson method not corrected for continuity [18]. Chi-square tests were used to compare LF prevalence between sexes and age groups, as well as the computed threshold of infection prevalence below which transmission is likely no longer sustainable, so-called critical cut-off threshold, against the observed proportion of ICT positive cases.

### Ethical considerations

This study was conducted as part of the action plan of the national program to eliminate lymphatic filariasis in Cameroon. Ethical clearance was granted by the Cameroon National Ethics Committee for Human Health Research (N°2014/09/491/CE/CNERSH/SP). Before enrolment, the objectives and schedule of the study were explained to the eligible population and individuals willing to participate signed two inform consent forms, and kept a copy. The second copy was stored at the Centre for Research on Filariasis and other Tropical Diseases. Even after minors assenting, the approval of their parents or legal guardians was necessary before any procedure. Each enrollee was assigned a unique identifier and his data analyzed anonymously. Positive cases were referred to CDDs and health officers for a close follow-up during next treatments, and their parents or legal guardians warned about the situation to further insure a better compliance. Although no guidelines are given in the TAS manual [2], the number of positive cases- that can be up to 18 as was the case in the present study -, appears as a real concern in a context where MDA has to be halted if the EU passes TAS. In this context, we have recommended to treat these rare positive cases with ivermectin during the MDA campaign plan just after the survey, then by a long course of doxycycline (4–6 weeks) when they get above 8 years and MDA no longer available.

## Results

### Demographic data

A total of 97 communities (EAs) were surveyed in the three EUs, and 5292 children (48.9% females) examined. These children were aged 5–8 (median age: 6) years old. Among the 5292 enrollees, 4171 (78.8%) were aged 6–7 years old (initial target), a small proportion being aged 5 (11.8%) or 8 (9.4%) years old. A total of 1595 children were examined in EU#1, 1919 in EU#2 and 1778 in EU#3, the expected sample size being reached in all the three EUs (Table 1).

**Table 1:** Number of enumeration areas visited, number of children examined, sex ratio and prevalence of ICT by evaluation unit.

Evaluation Unit	No villages visited	Expected sample size	No children examined	Sex-ratio (M/F)	No ICT positives cases (%)	95% CI
Mokolo	30	1552	1595	1.06	2 (0.13)	0.04–0.46
Ngong / Poli	34	1556	1919	0.99	11 (0.57)	0.32–1.02
Rey-Bouba / Tcholliré	33	1556	1778	1.09	8 (0.45)	0.23–0.89
**Overall**	**97**	**4664**	**5292**	**1.04**	**21 (0.40)**	**0.26–0.61**

### CFA prevalence

Prevalence of *W*. *bancrofti* circulating antigens, assessed using ICT card test, was equal to 0.13% (95% CI: 0.04–0.46) in EU#1, 0.57% (95% CI: 0.32–1.02) in EU#2, and 0.45% (95% CI: 0.23–0.89) in EU#3 (Table 1). The overall prevalence was 0.40% (95% CI: 0.26–0.61), with 80.95% positive cases aged 6–7 years old. The prevalence of LF was similar, both between age groups and sexes (*p* > 0.7408). The spatial distribution of positive cases was in general over-dispersed (both among health districts and EAs), except in the EU#2 where 8 children (1.07%; 95% CI: 0.54–2.10) with *W*. *bancrofti* circulating antigens were found in the Ngong health district, 6 of them belonging to two EAs.

### Critical cut-off values

The total number of LF positive cases was 2 in EU#1, 11 in EU#2 and 8 in EU#3, all below the critical cut-off threshold (18 in each EU) generated by the Survey Sample Builder. As compared to the threshold of infection prevalence below which transmission is likely no longer sustainable, the proportion of positive cases was significantly lower in the EU#1 (Chi-square = 12.68; *p* = 0.0004) and EU#3 (Chi-square = 5.27; *p* = 0.02), but not significantly lower in EU#2 (Chi-square = 3.48; *p* = 0.06).

## Discussion

In Cameroon, MDA against LF, using the combination of ivermectin and albendazole, started in 2008 in the North and Far North Regions. In 2014, five health districts (Mokolo, Ngong, Poli, Rey-Bouba and Tcholliré) completed six MDA rounds, and successfully passed the assessment of impact of MDA on LF infection after the fifth round of MDA (post 5^th^ MDA survey). The objective of the present study was thus to check whether the transmission of the disease has been successfully halted.

Based on historical data [10], sentinel sites’ survey data and/or kriging data [9,12], the North and Far North Regions were previously highly endemic for LF, and were reported among the most prevalent over the country. In 2014, LF prevalence observed in each of the three EUs investigated—in average equal to 0.40% (95% CI: 0.26–0.61)—was significantly lower than the threshold below which the transmission of the disease can no longer be sustained. Indeed, it was accepted that in areas where *W*. *bancrofti* is endemic and *Anopheles* or *Culex* is the principal vector, this target threshold must be < 2% antigenaemia prevalence [2]. In Cameroon, LF entomological data are very scanty but malaria data can be informative. Although *Anopheles gambiae* and *Anopheles funestus* have been found naturally infected with *W*. *bancrofti* [10], malaria entomological data have shown that the most abundant vectors in the Northern Cameroon are from genera *Anopheles* and *Culex* (Nwane, personal communication).

The number of positive antigenaemia cases observed in each of the three EUs surveyed was below the critical cut-off generated by the SSB (18 CFA positive cases), indicating that the area successfully “passed” TAS, and a cessation of MDA in the constituting communities should be envisioned. Indeed, the sample sizes and critical cut-off values were chosen so that an EU has (i) at least a 75% chance of passing TAS if the true prevalence of antigenaemia is 1.0% (half the target level if the vector is *Anopheles* or *Culex*), and (ii) no more than about a 5% chance of passing (incorrectly) TAS if the true prevalence of antigenaemia is ≥2.0% [19,20]. The importance of transmission assessment surveys as an evaluation tool for stopping MDA have been previously demonstrated in a multicenter evaluation using different approaches or study designs [21]. Moreover, the validity of TAS was also proven in long term post-MDA surveillance, although complementary test (antibody and xenomonitoring) appear of interest to ascertain the interruption of transmission during post-MDA surveillance [22–24].

It is important to notice that the interruption of transmission was achieved despite the fact that in some health districts, the effective treatment coverage was not reached for one or two rounds, although globally higher than 65% (S1 Table). TAS was considered for these health districts for three main reasons: (i) long lasting insecticidal nets (LLINs) have been distributed in the study area in the framework of malaria control program activities. Indeed, between 2003 and 2010, more than two millions LLINs have been distributed to pregnant women and children under 5 years old. In the framework of LLINs universal coverage for the control of malaria, a total of 21,028,770 LLINs have been distributed in the entire country in 2011 and 2016 (with 73% coverage in 2011 and 88% coverage in 2016), on the basis of one LLIN for every 2.2 households [25]. The impact of LLINs on prevalence and intensity of LF infection is now widely accepted [26,27], and it was shown that a sustained reduction in LF prevalence can be reached in spite of missed rounds of MDA [28]. In addition to these efforts related to the known usefulness of LLINs, the relatively poor compliance observed at the beginning of this large scale control strategy against malaria, and to some extent against LF, was improved over time thanks to communication and sensitization of populations [29]. It seems worth to mention that although insecticide resistance has been reported in several foci in Cameroon, it was demonstrated that LLINs might still offer some protection against the resistant *Anopheles gambiae* s.l. populations in northern Cameroon [30]. (ii) Ivermectin has been widely distributed in the study area since 1987 (Fig 2). Indeed, in 1987–1989, limited MDA campaigns were organized in the framework of a phase IV trial of ivermectin conducted in the Vina Valley located in the North Region of Cameroon [31]. In 1992, the Ministry of Health (MoH) and the River Blindness Foundation (RBF) began to broaden distribution of ivermectin, with the assistance of non-governmental developmental organizations (NGDOs), through mobile teams/outreach approach [32]. Since 1997–1998, the African Program for Onchocerciasis Control (APOC) joined the coalition to support annual delivery of ivermectin through community-directed treatment with ivermectin (CDTI) [33]. Although ivermectin is not macrofilaricidal, it is highly microfilaricidal and repeated treatments might have significantly contributed to the interruption of LF transmission [34,35]. Moreover, it was demonstrated that the transmission can be interrupted earlier than expected in areas previously treated for onchocerciasis [36]. (iii) Last but not the least, the prevalences in the study areas were relatively low when MDAs against LF began, suggesting that in such context, LF endemicity can be quickly lowered to level under which transmission cannot be sustained.

After six years of MDA (ivermectin in combination with albendazole), the transmission of LF was interrupted in five IUs (Mokolo, Ngong, Poli, Tchollire and Rey-Bouba heath districts) of the North and Far North Regions. These results support the cessation of MDA in these IUs, but this decision needs further thinking. It was demonstrated that MDA can be safely stopped in some but not all local government areas of Plateau and Nasarawa States in Nigeria [37], suggesting that the cessation of MDA can be feasible in the IUs investigated in northern Cameroon, even if the transmission of LF might be ongoing in the neighboring IUs. This is likely in accordance with the focal LF transmission that might be occurring in Cameroon. Also, the LF prevalences were relatively low at the beginning of MDAs, and the neighboring EUs has already completed at least four effective rounds of MDA when mass treatments can be halted as a consequence of transmission interruption.

However, epidemiological surveys conducted in northern Cameroon in 2008–2010 showed that mass ivermectin distributions had significantly lowered prevalence and intensity of onchocerciasis, but the transmission of the disease was yet to be interrupted [33]. In such circumstances where onchocerciasis transmission is still ongoing in these (and the neighboring) IUs, the interruption of treatments (IVM + ALB) might need further thinking. It is accepted that in areas where onchocerciasis is endemic, ivermectin can be used solely after interruption of LF transmission but this might be challenging while conducting surveillance activities to investigate potential recrudescence of LF. Another important challenge to take into account is the endemicity of soil transmitted helminthiasis (STH) since both IVM and ALB are effective against the parasites responsible of these diseases, especially in areas where STH control is not performing well. In such circumstances, it appears useful to investigate the situation of onchocerciasis and STH, especially now rapid diagnostic tests are being releasing for these diseases. This will help taking the decision about stopping MDA not only according to the evidence of LF transmission interruption, but also to the situation of STH and onchocerciasis in the selected EU. These additional data, collected in an integrated manner during TAS surveys, will be really cost effective and provide more insights in decision making.

## Supporting information

S1 Checklist STROBE checklist. (DOCX)

S1 Dataset Database for lymphatic filariasis transmission assessment survey in three evaluation units in northern Cameroon. EU: evaluation unit; IDN: identification number; ICT: immunochromatographic test; TAS: transmission assessment survey; Neg: negative; Pos: positive; M: Male; F: female.
(XLSX)

S1 Table Population and therapeutic coverage reported in the target implementation units (IUs) between 2008 and 2013. (DOCX)

S2 Table Population description of the targeted evaluation units. (DOCX)
