# Supplementary material for: First evidence of lymphatic filariasis transmission interruption in Cameroon: Progress towards elimination
Source: PLoS Negl Trop Dis. 2017 Jun 29;11(6):e0005633. doi: 10.1371/journal.pntd.0005633 (PMC5490934; doi:10.1371/journal.pntd.0005633)
Supplement: S1 Table — (DOCX) [file pntd.0005633.s003.docx]

**S1 Table. Population and therapeutic coverage reported in the target implementation units (IUs) between 2008 and 2013**

| **Regions** | **IU** |  | **Population (therapeutic coverage)** | | | | |
| --- | --- | --- | --- | --- | --- | --- | --- |
|  |  | **2008** | **2009** | **2010** | **2011** | **2012** | **2013** |
| Far-North | Mokolo | 173,598 (80.3%) | 179,875 (78.2%) | 187,387 (86.0%) | 208,876 (79.0%) | 214,307 (79.9%) | 219,879 (72.9%) |
| North | Ngong | 134,361 (74.3%) | 132,978 (81.4%) | 153,680 (64.0%) | 186,661 (42.0%) | 191,514 (78.1%) | 196,493 (76.5%) |
| North | Poli | 66,857 (63.0%) | 54,428 (81.3%) | 47,519 (84.0%) | 79,258 (57.0%) | 81,318 (74.9%) | 83,432 (74.8%) |
| North | Tchollire | 93,554 (77.6%) | 107,642 (69.0%) | 105,541 (79.0%) | 131,790 (71.0%) | 135,217 (75.5%) | 138,733 (81.0%) |
| North | Rey-Bouba | 80,430 (57.4%) | 79,523 (69.7%) | 111,537 (64.0%) | 100,720 (72.0%) | 103,338 (78.1%) | 106,025 (73.2%) |
