# Supplementary material for: First evidence of lymphatic filariasis transmission interruption in Cameroon: Progress towards elimination
Source: PLoS Negl Trop Dis. 2017 Jun 29;11(6):e0005633. doi: 10.1371/journal.pntd.0005633 (PMC5490934; doi:10.1371/journal.pntd.0005633)
Supplement: S2 Table — (DOCX) [file pntd.0005633.s004.docx]

**S2 Table. Population description of the targeted evaluation units**

| **EU** | **IU** | **EA** | **Total population** | **No children aged**  **6 - 7 years old** | **Average No children aged**  **6 - 7 years old per EA** |
| --- | --- | --- | --- | --- | --- |
| EU#1 | Mokolo | 133 | 219,879 | 21,988 | 161 |
| EU#2 | Ngong / Poli | 274 | 279,926 | 27,993 | 51 |
| EU#3 | Tcholliré / Rey-Bouba | 222 | 244,758 | 24,476 | 55 |

The data in this table were collected from census data of community distributors (CDDs) performed prior 2013 MDA, and the 2011 report of the National Institute of Statistics. EU: evaluation unit; IU: implementation unit; EA: enumeration area; No: number of.
